# Relationshi̇p between atrial fibrillation and coronary bypass surgery

**DOI:** 10.12669/pjms.303.4762

**Published:** 2014

**Authors:** Sedat Ozcan

**Affiliations:** 1 Sedat Ozcan, Department of Cardiovascular Surgery, Canakkale Onsekiz Mart University, Faculty of Medicine, Canakkale, Turkey.

**Keywords:** Atrial Fibrillation, Coronary bypass

## Abstract

***Objective:*** Atrial fibrillation (AF) is the most common arrhythmia seen after coronary artery surgery. The purpose this study was to determine incidence of AF that develops after coronary surgery and the factors affecting its development.

***Methods:*** Four hundred and forty eight patients who had coronary bypass surgery between February 2007 and September 2011 in the Cardiovascular Surgery Clinic were included in the study. Patients with history of chronic renal failure, redo coronary bypass surgery, valvular disease, thyroid disease, ventricular aneurysm and treatment with beta-blockers were excluded from the study of EF.

***Results:*** Two hundred and ninety nine patients were male and 149 were female and their age varied between 38 and 85 and their mean age was 61±5. Surgery was performed on beating heart on 178 patients and the others were operated with cardiopulmonary bypass (CPB). Advanced age, male sex and history of hypertension was more in AF developed patient group. There was no difference between AF developed group and the other group in terms of diameter of left atrium, ejection fraction (EF), CPB time and cross clamp time.

***Conclusion:*** Advanced age, male sex, hypertension and prolonged P-wave duration are the factors that are associated with AF after coronary surgery. Avoidance of CPB does not decrease AF development.

## INTRODUCTION

Atrial fibrillation (AF) that develops in the postoperative period is mostly temporary and spontaneously returns to sinus rhythm. But because high ventricular response dependent to AF may lead to decrease in cardiac output, hypotension, congestive heart failure and there is no atrial contraction and thromboembolic events may occur, postoperative AF is the most frequent reason of mortality after coronary surgery and may lead to deterioration of hemodynamics and increase in thromboembolic events.^1^

The treatment of AF developed after coronary surgery initially only consists of control of ventricular rate. If AF does not recover and hemodynamic deterioration occurs, pharmacological or electrical fibrillation is preferred. It is also suggested that AF disturbs flow in bypass grafts.

The purpose of this study was to evaluate the factors affecting AF development after coronary surgery.

## METHODS

 A total of 448 patients who had coronary bypass surgery between February 2007 and September 2011 were included in the study. Patients with previous coronary bypass operation, valve replacement at the same time, ventricular aneurysm surgery, chronic renal failure, impaired thyroid function were excluded. Coronary bypass surgery was performed on beating heart on 178 patients and with coronary bypass on the others. 

Left anterior mini-thoracotomy incision was used in two of the patients who had bypass surgery on beating heart and median sternotomy incision was used in the others. Median sternotomy incision, aortic arterial and two-stage venous cannulation was applied in operation with CPB. Myocardial protection was established by antegrade-retrograde combined cardioplegia. Proximal anastomosis was performed under cross clamping in patients who had CPB. But proximal anastomosis was performed using parsial occlusion clamp in patients who had bypass surgery on beating heart.

AF frequency was calculated to include the first postoperative 5 days. Arrhythmia analyses were carried on with ECGs with 12 derivations taken during arrhythmia and continuous ECG monitorization of patients sensitive to arrhythmia during service cares of patients monitored continuously with bed-side monitors for 2-4 days in intensive care unit. AFs that continued more than 15 minutes were evaluated. AF was defined as absence of P-wave before each QRS complex and presence of irregular ventricular rate.

Age, gender, hypertension, diabetes, changes in preoperative ECG (duration and amplitude of P wave), congestive heart failure, left ventricular ejection fraction (EF), size of left atrium, CPB time, cross clamp time was compared between AF developed and not developed patients.

Data were presented as mean ± standard deviation. Student-t test was used for statistical analysis. P was considered as significant when smaller than 0.05.

## RESULTS

One hundred and ninety four patients were male and 52 were female and their age varied between 38 and 85 and mean age was 61±5. Eighty seven patients (19.41%) developed AF. AF developed in 25 of 178 patients (14.06%) who had bypass surgery on beating heart and 62 of 267 patients (24.3%) who had bypass surgery with CPB. AF developed between postoperative 2^nd^ and 5^th^ days (mean 47.8± 11.4 hours). The mean age of patients with AF was 58±9 and mean age of patients who did not develop AF was 55±7 and it was statistically significant (p<0.05). Ratio of male patients and hypertension history was higher in the group with AF than the group without AF but there was no statistically significant difference with regards to ratio of diabetic patients (Table-I). There was no statistically significant difference between two groups with regards to size of left atrium, ejection fraction (F) and cross clamp time (p>0.05). Duration of stay in intensive care unit and mortality was higher in the group with AF (p<0.05).

Duration of P-wave was significantly higher in DI, DII and aVF derivations in patients with AF. But there was no difference in the other derivations. Amplitude of P-wave was significantly higher in VI and VII derivations in group with AF. But there was no significant difference in the other derivations.

While mean creatine phosphokinase M-B (CPK-MB) was 21.7±15.8 microgram/l in patients with AF it was 23.5±16.8 microgram/l in the other group and it was statistically significant (p>0.05). Fifteen patients who developed postoperative AF were treated. The others recovered spontaneously.

## DISCUSSION

Although the reason of postoperative AF developed in patients who had coronary bypass surgery is not known, multiple risk factors and triggering events were considered responsible. It is suggested that CPB is a major risk factor.^2^ Besides, it is claimed that atrial manipulations, cannulation, cardioplegic arrest, prolongation of cross clamp duration, electrolyte disorders, temporary ischemia, perioperative trauma, epicardial inflammatory reactions, euthyroid syndrome, cessation of beta blockers due to operation, advanced age, male sex, hypertension, myocardial infarction history, respiratory problems, creation of hematoma, hypoxia, hypovolemia sepsis, weak protection of atrial tissue, release of systemic mediators plays role in the development of AF. It is asserted as a postulate that increase of catecholamines in circulation is an important pathogen for triggering AF.^3^^-^^6^

AF is most frequently seen due to cessation of drugs of the patients who use beta blockers before surgery. This is caused by release of catecholamine in postoperative period. Triggering factors such as atrial wall tension, ischemia inflammation or autonomic nervous system imbalance cause changes in conduction and refractory that form a trend for AF. Age and structural changes related to age such as dilatation in atrium, loss of nodal fibres, muscle atrophy, increase of fibrosis tissue and fat tissue in sinus node and accumulation of local interstitial amiloid increase tendency to postoperative AF. These structural changes may explain why AF ratio is high in old patients in our series. These changes cause changes in local atrial refractory periods. This is called refractor dispersion. Disruption of uniformity may cause AF together with triggering factors.^7^

P-wave duration measured preoperatively is suggested to be an independent risk factor for estimating postoperative AF development. Because prolongation of P wave duration shows left atrial expansion or prolongation of intraatrial conduction as in our series, it is an important substrate in the development of AF.^8^

**Table-I:** Clinical characteristics of patient groups with and without AF

	*Patient group with AF*	*Patient group without AF*
Age (year)	58±9	55±7
Gender (male/female)	64/23	235/126
Hypertension (number of cases and %)	23 (26.4%)	95 (26.3%)
Diabetes mellitus (number of cases and %)	15 patients (17.2%)	63 patients (17.3%)
Width of left atrium (mm)	38±6	36±6
Ejection fraction (%)	60±11	56±13
Perfusion duration (minute)	86±24	83±19
Cross clamp time (minute)	59±19	55±16
Duration of stay in the intensive care unit (hour)	61±13	122±23
Mortality(number of cases)	6 (6.89%)	11 (3.23%)

Advanced age is related with increase in level of norepinephrine in circulation.^9^ These patients are more sensitive to cessation of beta blockers in postoperative period and are supported by more frequent postoperative supraventricular beats and postoperative supraventricular increase. Increase of sympathetic stimulation secondary to postoperative trauma is also important.^8^

It is important to protect atrium with cardioplegia during surgery to decrease postoperative AF. Atrium withdraws less cardioplegic solution than ventricles. These electrophysiological changes in atrium conduction cause more postoperative supraventricular arrhythmias. Ultrastructural and morphometrical studies of mitochondrion obtained from the biopsy samples of patients who had cardiac surgery showed that atrium was less protected than ventricles.^10^ The mechanism of AF is not defined yet.

AF incidence increases 1.7 fold in every decade due to enlargement of cardiac cavity and development of fibrosis as a result of ageing.^11^ If intrapericardial dissection is performed during resection of lung, AF develops more frequently. If pericardial fluid accumulates in patients who had heart valve surgery, AF ratio increases.^12^

Incidence of acute AF is observed between 15-45%. The incidence was 19.41% in our series. Although AF rarely causes mortality it increases postoperative morbidity. Especially due to changes in ischemic cardiac disease, cardiac output and high ventricular rate, increase in heart workload and loss in atrial contractions are adverse effects.^13^ Postoperative AF is seen 2-5 days after operation. AF is well tolerated in many of the patients and 96% recovers spontaneously 6-7 weeks after operation as in our series. However, loss of atrial contractions may not be tolerated in patients with diastolic dysfunction. Therefore patients with diastolic dysfunction should be treated.

AF may increase heart rate and central venous pressure and decrease mean arterial pressure and significantly decrease cardiac index; besides it may decrease blood flow in all grafts especially in internal mammarian artery graft. It is rarely seen immediately after operation. The reason why postoperative AF is observed 2-3 days after operation and not very much observed in early period is still not known. The probable explanation of this is the time needed for development of the said triggering factors and cause of electrophysiological abnormalities.^8^ Because diastolic dominance of IMA graft will disappear in the late period, postoperative AF will not have a significant effect. But if AF develops in early period it must be treated because it will affect graft flow and hemodynamics.^14^

AF is the most common arrhythmia in the society. Under normal conditions it is seen in 2.8% and 5% of the population respectively between age of 40 to 70 years and over 65 years. It dramatically increases after heart surgery. AF is seen in 35-40% and 65% respectively after coronary surgery and heart surgery.^15^ In another study it was stated that postoperative AF was seen in 4.7% and 35% of patients respectively below age of 40 and over 70.^8^ We consider that postoperative AF will increase as older population increases who has coronary surgery.

Development of persistent AF in patients older than 80 is 5 times more than development in young patients. 2.5 times more seizure is seen in patients who developed postoperative AF when compared with patients with sinus rhythm.^16^ AF incidence is three times more in patients with stroke attack history. Stroke rate is 13 times more in patients with AF in hospital. A possible explanation of development of persistent AF is the electrophysiological remodelling of AF.^11^ Perioperative cardiac enzyme levels are a little higher in patients who had bypass on beating heart (4.8%). This is possibly related to intraoperative regional myocardial ischemia.^7^

Fragmented and prolonged atrial activation is shown during sinus rhythm with intracardiac records only in patients with proximal AF. This finding is related to existence of arrhythmia re-entry mechanism of conduction delay regions. P-wave duration significantly increases in patients who developed AF after coronary surgery. It is possible that structural changes developed due to hypertension play an important role in the occurrence of arrhythmias together with hypertension. It may act as a substrate for the changes that occurred in hypertrophied heart and fibrosis reentry arrhythmia.^16^ High AF ratio in patients with hypertension in our series may be related to structural changes due to hypertension.

Age, male sex, hypertension, increase of P-wave duration in derivations DII, DIII and aVF in preoperative electrocardiographies, increase of P-wave amplitude in derivations VI, VII increased incidence of AF in our series after coronary surgery. Performance of heart surgery without use of CPB, diabetes, cross clamp time, perfusion time, EF and diameter of left atrium did not quite affect AF development in our series.
